# Efferocytosis in dendritic cells: an overlooked immunoregulatory process

**DOI:** 10.3389/fimmu.2024.1415573

**Published:** 2024-05-21

**Authors:** Yanyan Ma, Tangxing Jiang, Xun Zhu, Yizhou Xu, Ke Wan, Tingxuan Zhang, Miaorong Xie

**Affiliations:** ^1^ Department of Emergency and Critical Care Center, Beijing Friendship Hospital, Capital Medical University, Beijing, China; ^2^ Department of Emergency Medicine, Qilu Hospital of Shandong University, Jinan, China

**Keywords:** dendritic cell, apoptotic cell, efferocytosis, immune regulation, antigen cross-presentation, Treg differentiation

## Abstract

Efferocytosis, the process of engulfing and removing apoptotic cells, plays an essential role in preserving tissue health and averting undue inflammation. While macrophages are primarily known for this task, dendritic cells (DCs) also play a significant role. This review delves into the unique contributions of various DC subsets to efferocytosis, highlighting the distinctions in how DCs and macrophages recognize and handle apoptotic cells. It further explores how efferocytosis influences DC maturation, thereby affecting immune tolerance. This underscores the pivotal role of DCs in orchestrating immune responses and sustaining immune equilibrium, providing new insights into their function in immune regulation.

## 1 Introduction

DCs are unequivocally central to the regulation of host immune responses. Recent advancements have highlighted the pivotal role of DCs in efferocytosis—a finely tuned mechanism where apoptotic cells are orderly cleared without triggering inflammatory responses. Dysregulation of efferocytosis is closely linked to the progression of various diseases, including autoimmune disorders (1), cancer (2), and chronic inflammation (3). While numerous studies have focused on the role of DCs in antigen presentation and the initiation of immune responses (4, 5), their unique contributions to efferocytosis and its profound impact on immune modulation remain to be fully elucidated.

This review synthesizes recent discoveries in DC-mediated efferocytosis, highlighting the distinct immunological functions and traits of DCs in this process. We explore the involvement of various DC subsets in efferocytosis, identifying and analyzing the ‘eat me’ signal molecules involved in DC-mediated efferocytosis. It is acknowledged that not all ‘eat me’ signals facilitate this process universally across phagocytes. The review distinguishes between signals that mediate efferocytosis in both macrophages and DCs, those unique to DC efferocytosis, and those exclusively involved in macrophage-driven efferocytosis, thereby shedding light on the selective mechanisms of efferocytosis in DCs. Additionally, we examine how DCs, through the efferocytosis process and subsequent antigen cross-presentation, contribute to the modulation of immune responses. The review further delves into how DC-mediated efferocytosis can cultivate immune tolerance by influencing DC maturation, thereby underscoring the critical role of DCs in fine-tuning immune responses and maintaining immunological balance.

## 2 Efficient clearance of apoptotic cells

In the human body, a remarkable process unfolds daily where hundreds of billions of cells undergo programmed cell death, leading to the generation of numerous apoptotic cells (6). This occurrence, despite its vast scale, remains rarely observed within the body due to the efficient clearance by phagocytes (7), a phenomenon aptly described by scientists as the “burial” of apoptotic cells, a process known as efferocytosis. The term “efferocytosis” was coined in 2006 by Peter Henson and his team, described the specialized phagocytosis of apoptotic cells (8). The cells responsible for efferocytosis encompass not only the professional phagocytes like macrophages and DCs but also include ‘non-professional’ phagocytes such as retinal pigment epithelial cells (9).

This process is distinct from the broader spectrum of phagocytosis, particularly in its mechanisms and biological implications. Traditional phagocytosis involves the identification and engulfment of pathogens by phagocytes through Toll-like receptors (TLRs) that recognize pathogen-associated molecular patterns (PAMPs), or through complement and Fc receptors when pathogens are opsonized. In contrast, efferocytosis employs specific receptors on phagocytes that detect “eat me” signals presented on the surface of apoptotic cells, such as members of the TIM and TAM receptor families (10).

From a biological standpoint, efferocytosis is characterized by its non-inflammatory and non-immunogenic nature, contrasting with the inflammatory response triggered by conventional phagocytosis of pathogens. The engulfment of apoptotic cells through efferocytosis does not incite the production of pro-inflammatory cytokines; rather, it results in the secretion of anti-inflammatory mediators that facilitate the resolution of inflammation (11).

Morphologically, the process of efferocytosis is distinguished by the active deformation of the phagocyte’s cell membrane and the creation of membrane ruffles, which serve to envelop and ingest apoptotic cells (12, 13). This contrasts with the engulfment of pathogens via complement receptors and FcRs, where the pathogens appear to “sink into” the phagocytes (14).

### 2.1 Molecular mechanisms and phases

Efferocytosis plays a pivotal role in maintaining tissue homeostasis and immune response regulation. This highly orchestrated process involves several critical steps: the identification, recognition, engulfment, and degradation of apoptotic cells. Below, we present a refined discussion of these steps, emphasizing the complex molecular interactions and signaling pathways involved.

### 2.2 Recognition and binding

In the initial phase, apoptotic cells release “Find-me” signals to attract phagocytes. These signals comprise a range of molecules such as sphingosine-1-phosphate (S1P) (15), CX3C chemokine ligand 1 (CX3CL1) (16), lysophosphatidylcholine (LPC) (17), and nucleotides including ATP and UTP (18). The collaborative effect of these signals effectively recruits phagocytes, facilitating the prompt removal of apoptotic cells.

Concurrently, apoptotic cells present “Eat Me” signals on their surface, promoting efferocytosis. Predominant among these signals is phosphatidylserine (PtdSer), complemented by molecules like calreticulin, ICAM-3, complement proteins, and alterations in cell surface charge and glycosylation patterns (18, 19). PtdSer’s presence is particularly notable as it marks a significant change on the surface of dying cells, distinguishing them from healthy cells, which exhibit “Do not eat me” signals such as CD47, CD24, CD31, CD46, and MHC class I molecules to avoid efferocytosis (20–24).

Phagocytes detect apoptotic cells via “Eat Me” ligands recognized by specific receptors on their surface. Key receptors include the CD300 family (CD300a、CD300b and CD300f), T cell immunoglobulin and mucin domain (TIM) receptors (TIM-1, TIM-3, and TIM-4), Stabilins (stabilin-1 and stabilin-2), brain angiogenesis inhibitor 1 (BAI1), receptor for advanced glycation end products (RAGE) (25), and scavenger receptors (SR-AI,SR-B1,CD36) (26). Additionally, some receptors like the TAM receptor tyrosine kinases (Tyro3, Axl, and Mertk) and integrins family require bridging molecules such as Growth-arrest-specific protein 6 (Gas6), Protein S, and Milk fat globule-EGF factor 8 (MFG-E8) for indirect recognition of the “eat me” signals Bridging molecules play a crucial role in the recognition process, selectively binding to receptors on phagocytes. Gas6, for instance, interacts with Tyro3, Axl, and Mertk, whereas Protein S only binds to Tyro3 and Mertk. Mfge8 and its homolog Developmental Endothelial Locus-1 (Del-1) target integrins to facilitate the recognition of PtdSer, further ensuring the efficient clearance of apoptotic cells (27, 28).

### 2.3 Engulfment

Phagocytes can internalize apoptotic cells whether through direct or indirect binding. This is because efferocytosis receptors, upon recognizing and binding apoptotic cells, can trigger various signaling pathways to activate small GTPases, including key members of the Rho family such as RhoA, Rac1, and CDC42. These GTPases play pivotal roles in modulating the actin cytoskeleton, as they alternate between their GDP-bound inactive states and GTP-bound active states. This alternation dictates the dynamics of actin filament organization, which is essential for phagocytosis. For example, αVβ5 can activate the p103cas-CRK-DOCK180 pathway, thereby triggering the activation of Rac1 (29). Mfge8 and Mertk can enhance the activation of Rac1 by αVβ5 (30, 31), while the trimeric complex formed by BAI1 with ELMO and DOCK180 can directly recruit Rac1, promoting the uptake of apoptotic cells (32). Additionally, SR-BI can activate PI3K and Rac1 by promoting Src phosphorylation and recruitment to the plasma membrane (33), and both LRP1 and stabilin-2 activate Rac1 through the adaptor protein GULP (34, 35). These interactions initiate cytoskeletal reorganization, thereby initiating cytoskeletal reorganization to facilitate the formation of efferosomes.

The level of GTP-bound Rac1 gradually increases during the recognition process of apoptotic cells, and Rac1 promotes the engulfment of apoptotic cells by phagocytic cells (29, 36–38). The activation pathway comprising CRKII, ELMO1, and DOCK180 serves as a pivotal conduit for Rac1 activation (38–41), Upon activation, Rac1 can interact with the WAVE regulatory complex, which in turn activates the actin nucleation complex Arp2/3, promoting the formation of branched actin network (42).— a critical event for actin polymerization. This polymerization is fundamental for phagocytic cup (the area where phagocytic cells contact apoptotic cells) formation (43–45), the nexus of phagocyte- apoptotic cells interaction, thereby facilitating the ultimate sequestration of apoptotic cells.

After the phagocytic binds to the apoptotic cells, RhoA is transiently upregulated just before the closure of the phagocytic cup, thereby delaying the phagocytic process (46). When RhoA is activated, it enhances the kinase activity of the Rho-associated coiled-coil-containing protein kinase (ROCK), leading to the phosphorylation of the myosin light chain (MLC) and promoting cellular contraction (47). This contraction may inhibit the extension of pseudopods and the closure of the phagocytic cup, which are crucial for the early stages of efferocytosis (47). The transient activation of RhoA ensures that the process does not proceed too quickly, thus giving the cell enough time to confirm whether the target cell truly needs to be engulfed. This mechanism not only ensures that cells only engulf those targets that need to be cleared but also prevents phagocytes from “overeating” (46, 48). RhoA also induces actin polymerization and the extension of pseudopods through distinct pathways that involve mDia and PI(4)P5K, along with further activation of ROCK, leading to the activation of Adducin. This regulates the stability of actin filaments, further supporting the formation of the phagocytic cup. As the process progresses, the level of RhoA gradually decreases, ensuring that phagocytosis can proceed normally, as inhibiting RhoA activity in this process can promote the phagocytosis of apoptotic cells (36, 37, 49). The fine regulation and dynamic changes of RhoA are key mechanisms in ensuring the effective clearance of apoptotic cells.

Cdc42 also contributes to actin polymerization and promotes the phagocytosis of apoptotic cells (50, 51). Cdc42 activates the effector proteins WASP (Wiskott-Aldrich syndrome protein) and N-WASP, which in turn promote the activation of the Arp2/3 complex, thereby inducing actin branching. This activation process leads to the formation of cellular pseudopodia (52–54) thereby enhancing cell migration capabilities and interaction with the environment. Studies utilizing overexpression of a dominant-negative form of Cdc42 and mice lacking the Cdc42 effector WASP have suggested a positive role for Cdc42 in the uptake of apoptotic cells (37, 55, 56).

### 2.4 Efferosomes processing

After actin polymerization forms the phagocytic cup and internalizes the apoptotic body, it then enters the degradation phase of the efferosomes. This degradation pathway parallels that of phagosomes, necessitating a sequential fusion with early endosomes, followed by late endosomes and ultimately lysosomes (57). Rab GTPases (Rab5, Rab7, and Rab17) and the autophagy-related protein LC3 play important roles in the formation and maturation of efferosomes. After phagocytic cup closes, actin disassembles, followed by the extension of microtubules. The microtubule-tip-associating protein, EB1, transports the guanine nucleotide exchange factor (GEF) Gapex-5 to the efferosomal membrane, increasing the ratio of GEF to GAP activity on the membrane, thereby activating and recruiting more Rab5 (58) Additionally, before the internalization of apoptotic cells, once phagocytes come into contact with apoptotic cells, they recruit Dynamin and Vps34 to the interface between the apoptotic cells and efferocytes. The interaction between Dynamin and Vps34 also facilitates the recruitment and activation of Rab5 to the efferosomes (59). Rab5-GTP further promotes the activation of Vps34, leading to the production of PtdIns(3)P on the surface of the efferosome (59). This PtdIns(3)P, in turn, recruits the effector protein Eea1 (Early Endosome Antigen 1), thereby enhancing the fusion of the efferosomes with early endosomes (58). At this point, the efferosomes begins to acidify (59). The next step involves the Mon1-Ccz1 complex, CED-1, and DYN-1 recruiting Rab7 to the efferosome and activating it (60, 61); Rab7, in turn, mediates the fusion of efferosomes with late endosomes and lysosomes (62, 63). The Rab7 effector protein Rab-interacting lysosomal protein (RILP) can interact with multiple subunits of the HOPS complex, including Vps41 and Vps39, and recruit them to late endosomes (59, 64, 65), triggering SNARE-mediated membrane fusion (66, 67). At this stage, the efferosome becomes increasingly acidic, leading to the activation of acidic proteases and nucleases, which then degrade the contents of the apoptotic cells (68). Additionally, Rab7 coordinates traffic between late endosomes and the lysosome, interacts with RILP to recruit the dynein-dynactin motor complex, and transports late endosomes towards centrosomes and the lysosome (62). leading to the formation of effero-lysosomes.

Microtubule-associated protein light chain 3 (LC3) also participates in the fusion of efferosomes with lysosomes, thereby promoting the maturation of efferosomes and more thorough degradation of their contents. Specifically, After the efferosome is formed, the LC3-II protein is recruited by autophagy family members and, following lipidation, becomes anchored to the efferosome membrane, a process known as LC3-associated phagocytosis (LAP). After the LC3-II protein binds to the membrane of the efferosome, the efferosome is typically renamed as a LAPosome. LAP is triggered by the recruitment of the class III PI3K complex, which includes components such as Rubicon, VPS34, Beclin-1, and VPS15 (69, 70), that facilitate the localization of PI3P to the efferosome. The localization of PI3P stabilizes the NOX2 complex to produce ROS. Both ROS and PI(3)P are necessary for LC3-II lipidation, thereby enabling its successful decoration on the efferosome. Subsequently, the LAPosome fuses with lysosomes, further enhancing the degradation of the contents (70).

**Figure 1:**
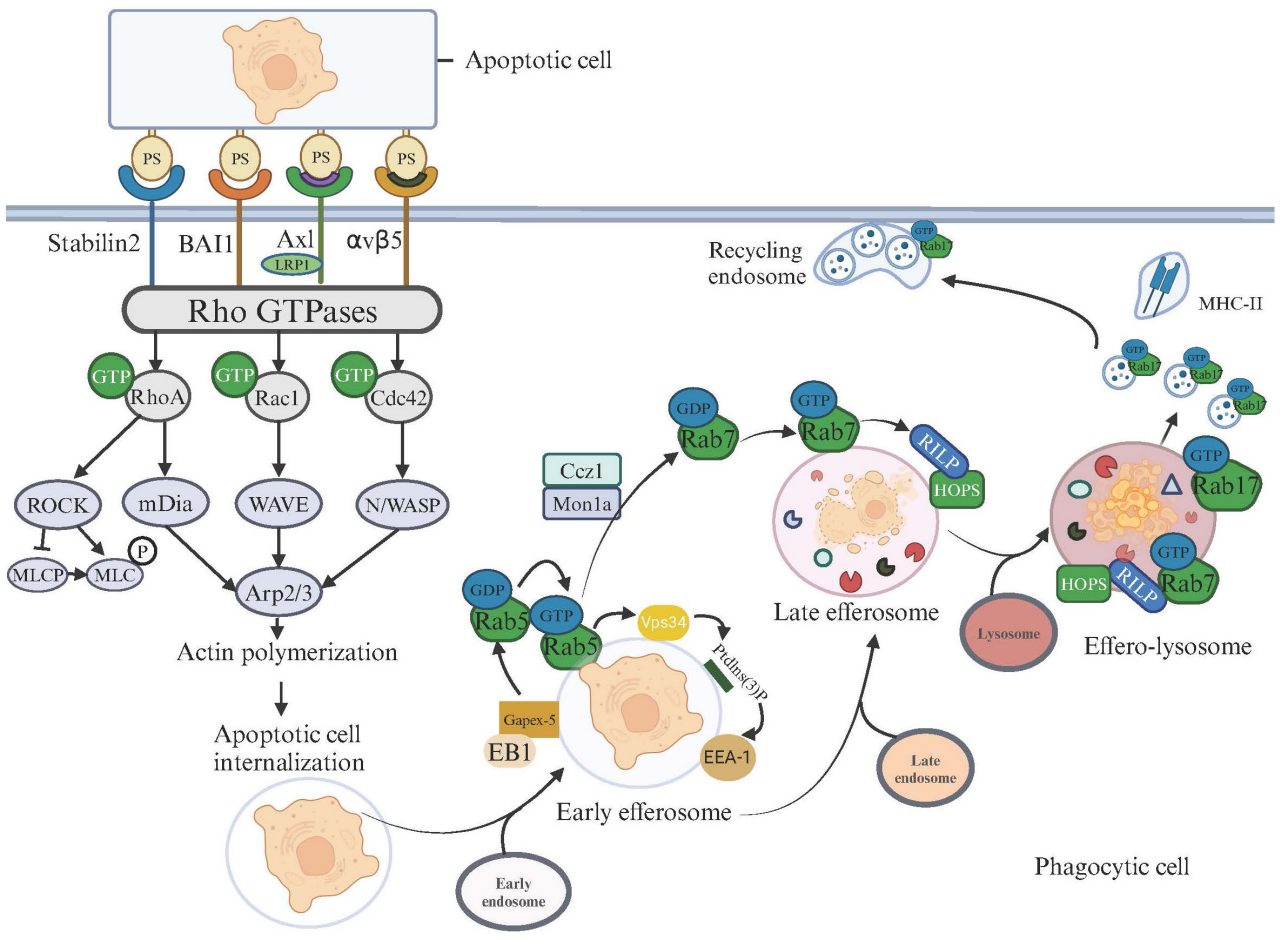
Apoptotic Cell Recognition, Internalization, and Degradation Pathways in Phagocytic Cells. This illustration depicts the signaling pathways activated for cytoskeletal rearrangement and apoptotic cell degradation following the recognition of apoptotic cells on the surfaces of phagocytic cells. Upon the binding of phosphatidylserine (PS) on apoptotic cells, surface receptors such as Stabilin2, BAI1, AXL, and integrin αvβ5 on phagocytic cells initiate signaling cascades. These cascades activate Rho GTPases, including RhoA, Rac1, and Cdc42, which subsequently trigger various downstream effectors. Activation of RhoA leads to the ROCK-mediated phosphorylation of MLC via MLCP, influencing actin polymerization, which is crucial for the formation of the phagocytic cup. Rac1 and Cdc42 facilitate actin assembly through mDia and the WAVE/Arp2/3 complex, respectively, enhancing the engulfment and internalization of apoptotic cells. After internalizing apoptotic cells, the maturation and degradation pathways commence. The microtubule-tip-associated protein EB1 transports the GEF Gapex-5 to the efferosome membrane, activating Rab5-GDP. The activated Rab5-GTP further promotes the activation of Vps34, leading to the production of PtdIns(3)P on the efferosome surface. This PtdIns(3)P subsequently recruits the effector protein Eea1 (Early Endosome Antigen 1), enhancing the fusion of efferosomes with early endosomes. At this point, the efferosome begins to acidify. The next step involves the Mon1-Ccz1 complex recruiting and activating Rab7 on the efferosome. Rab7 recruits the effector protein RILP, which in turn recruits and interacts with the HOPS complex, facilitating the sequential fusion of the early efferosome with late endosomes and lysosomes, ultimately forming the effero-lysosome. At this stage, the efferosome becomes increasingly acidic, activating acidic proteases and nucleases, beginning the degradation of apoptotic cell contents. Rab17 drives the division of effero-lysosomes into smaller vesicles and directs these vesicles to merge with recycling endosomes near the cell membrane, promoting material recycling and distancing from MHC-II.

Different from phagosome degradation, effero-lysosome degradation incorporates the involvement of Rab17 (71), which is a distinction in the mechanistic pathway. Propelled by the small GTPase Rab17, effero-lysosomes undergo division into smaller vesicles. Rab17 directs these Exosome-Derived Vesicles (EDVs) from the cell’s central to its peripheral regions. At the periphery, EDVs integrate with recycling endosomal compartments. This strategic relocation segregates EDVs from the Major Histocompatibility Complex (MHC) class II antigen-loading compartments (72). Consequently, this segregation prevents the exhibition of antigens originating from apoptotic cells, emphasizing a critical immunoregulatory mechanism. Pathways of Apoptotic Cell Recognition, Internalization, and Degradation are depicted in **Figure 1**.

### 2.5 Residue removal and resolution of response

The fusion of EDVs with recycling endosomal compartments enables the recycling of cellular components such as carbohydrates, amino acids, lipids, and nucleotides. These metabolites and other cellular components derived from the engulfed apoptotic cell can modulate functional responses in phagocytes (73–78).The digestive degradation mechanism of effero-lysosomes is a highly complex biological process involving the acidification of lysosomes and endosomes; their acidic environment is maintained by the continuous pumping of protons (H+) from the cytosol into the lysosomes or endosomes by V-ATPases (79, 80). This acidic environment is crucial for activating the various hydrolases required to break down cellular debris within effero-lysosomes. This enzymatic activity not only helps decompose cell fragments into reusable biomolecules, aiding in cellular maintenance and growth, but is also essential to prevent undigested cargo from improperly triggering intracellular signaling pathways, such as the cGAS-STING pathway triggered by undigested DNA, which in turn produces type I interferons (81), or the activation of lipid sensors of the peroxisome proliferator-activated receptors (PPAR) and liver X receptors (LXR) families (75, 76, 78) that orchestrate immune-tolerance associated programs. In murine macrophages, it has been observed that the identity of the engulfed cell has a significant impact on the functional programs triggered by efferocytosis, underscoring the highly precise decoding machinery underlying efferocytosis (82). Given the critical nature of this process in health and disease, future research efforts should focus on elucidating the transport and degradation mechanisms of efferosomes.

## 3 Efferocytosis by specialized DC subsets

### 3.1 Distribution and classification of DCs

**Figure 2:**
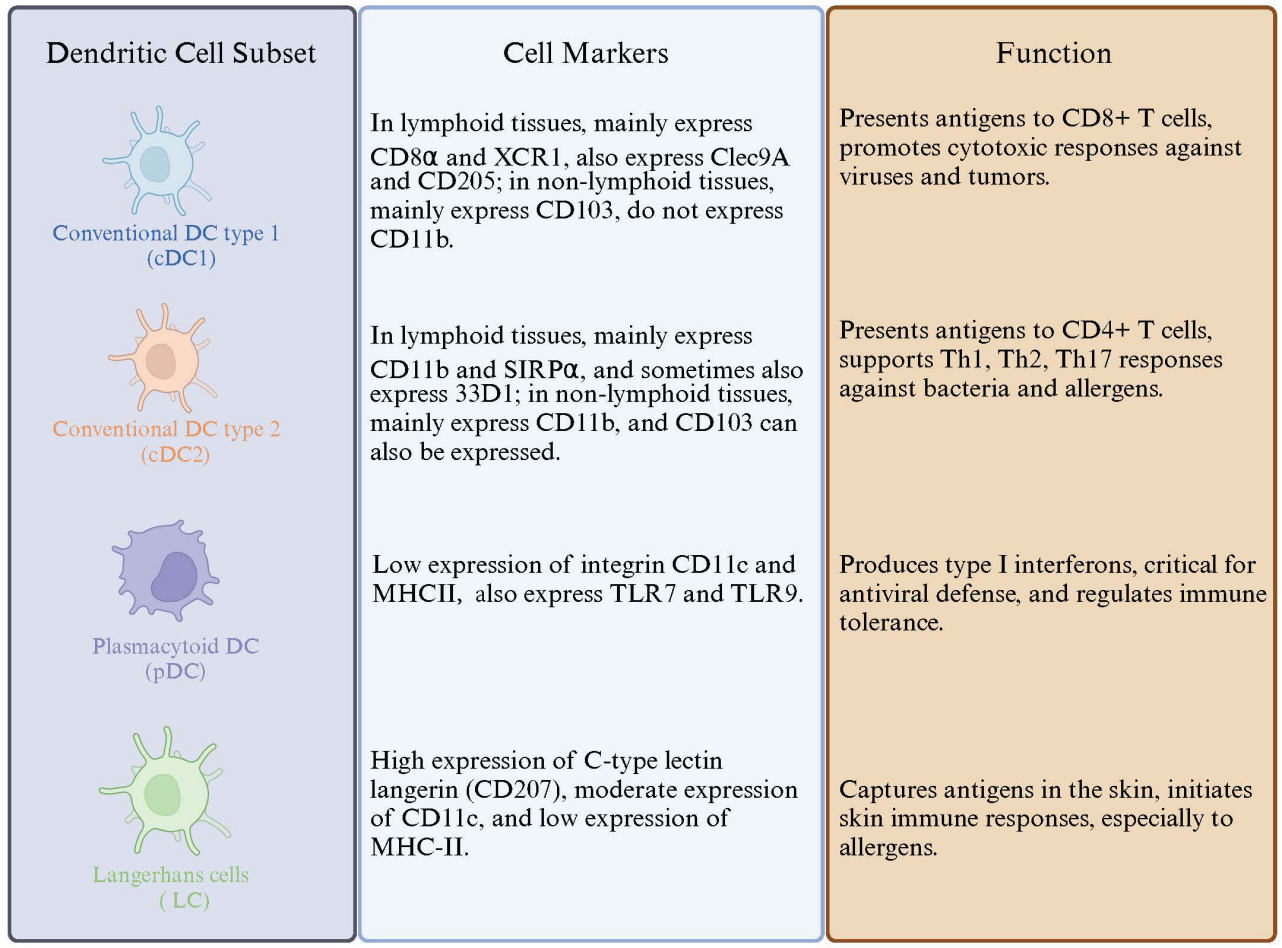
Dendritic Cell Subtypes and Their Markers and Functions. This figure illustrates the distinct subsets of dendritic cells, detailing their specific cell markers and functional roles in immune responses.

DCs are widely distributed in various organs and tissues of the human body except for the brain and eyeballs, commonly found in blood, skin mucosa, and lymphoid tissues, accounting for less than 1% of the total peripheral blood white cells (83). DCs are differentiated from lympho-myeloid hematopoietic stem cells. DCs are composed of several subsets, which include conventional DCs (cDCs, further distinguished as cDC1 and cDC2) and plasmacytoid DCs (pDCs) (84). DC subsets along with Their Markers and Functions are illustrated in **Figure 2**.

### 3.2 CD8+ cDC1

The cDC1 subset is distinguished by its capacity to cross-present exogenous antigens, facilitating the activation of CD8+ T cells (85). The genesis of cDC1 is regulated by the transcription factors BATF3 and IRF8 (86).

Further advancements in our understanding were propelled by the investigative efforts of Albert’s research team, which unveiled an additional functional facet of cDC1 subsets—their ability to engulf apoptotic cells (87). This phagocytic competence adds a new layer to the functional repertoire of cDC1, positioning them as pivotal players in maintaining immune homeostasis and in the orchestration of immune responses against pathogens.

The mechanism of action predominantly employed by cDC1 in the presentation of antigens from apoptotic cells involves MHC class I molecules (88). This discovery has spurred a series of investigative endeavors aimed at elucidating the processes governing DCs phagocytosis of apoptotic cells and the subsequent antigen presentation (89–92). Among these, a notable study conducted by Tomonori Iyoda et al. in Japan employed CFSE-labeled apoptotic spleen cells in mice to trace the phagocytic activity of DCs *in vivo* (93). The findings revealed a selective efferocytosis capacity within the DC subsets; while both CD8+ cDC1 and CD8- cDC2 subsets in the spleen were capable of engulfing latex beads, only the CD8+ cDC1 subset was proficient in phagocytosing the CFSE-labeled apoptotic spleen cells. This significant finding emphasizes that CD8+ cDC1 subsets have the superior capacity to phagocytose apoptotic cells compared to other types of DCs. Interestingly, this phenomenon is mirrored in the human immune system, where CD141+ DCs, akin to the murine CD8α+ cDC1 subset, exhibit the ability to phagocytose necrotic cells (94), further highlighting the conserved functional attributes across species. It is important to note that the majority of evidence concerning the efferocytic capabilities of DCs originates from studies conducted on mice.

Subsequent inquiries delved into the underlying reasons for the differential phagocytic abilities observed between CD8+DCs and CD8-DCs. A hypothesis emerged suggesting a potential linkage to the distinct efferocytosis receptors expressed by the respective DC subsets. In the murine spleen, DCs segregate into two primary subsets: CD8α+DEC205+DCs, localized within the T cell zones, and CD8α-33D1+DCs, situated in the red pulp and marginal zones (95). Notably, the CD8+ cDC1 subset is characterized by elevated levels of DEC205 expression, in contrast to the CD8-cDC2 subset, which lacks DEC205 expression (96–98). This led to investigations into DEC205’s role in mediating phagocytosis of apoptotic cells. However, the outcomes from studies using DEC205 gene knockout mice indicated that the absence of DEC205 did not impede the ability of the CD8+ cDC1 subset to phagocytose apoptotic cells (93), suggesting that despite its high expression, DEC205 is not the crucial factor in the efferocytosis activity of this subset. DEC205 acts as an endocytic receptor for phagocytosis, rather than as an efferocytic receptor for efferocytosis. It promotes the internalization and subsequent presentation of antigens (rather than apoptotic cells as antigens) among immune cells, thereby regulating immune responses (99). Subsequent studies using RT-PCR and microarray analysis have found that the expression of CD36 on CD8+DCs is significantly higher than on CD8-DCs, which may be a key factor contributing to the differences in their ability to engulf apoptotic cells (93, 100). The latest reliable evidence further confirms that CD36 indeed enhances the phagocytic capacity of DCs towards apoptotic cells (101).

### 3.3 CD103+ cDC1

A fraction of cDCs found in lymphoid and non lymphoid organs expresses the E-cadherin–binding integrin αE (CD103) (102, 103). The exploration of the ability of DC subsets to phagocytose apoptotic cells, has significantly advanced with contributions from the research teams led by Chun-Hong Qiu and Yasunobu Miyake (100). A notable discovery in this realm is the unique behavior and localization of CD103+CD207+DCs within the CD8α+cDC1 group in the spleen. Contrary to the conventional localization of CD8α+cDC1 subsets within T cell zones, these specific DCs exhibit a propensity to migrate to the marginal zones following the phagocytosis of blood-derived apoptotic cells.

This migratory pattern facilitates a specialized function of the CD103+DC subset in the spleen, where they preferentially engulf blood-derived apoptotic cells. Post-phagocytosis, these cells relocate to the T cell zones to engage in the cross-presentation of cell-associated antigens, thereby promoting antigen-specific immune responses. This behavior underscores the specialized role of CD103+DC subsets in the immune surveillance and response mechanisms, particularly in the context of apoptotic cell clearance and antigen presentation.

Further investigations have expanded the understanding of CD103+DCs beyond the spleen, revealing a genetic homology between lung tissue CD103+DCs and spleen CD8α+cDC1 (104, 105). This homology underpins the classification of lung CD103+DCs within the cDC1 subset, emphasizing the broader relevance of these cells across different anatomical sites. In the absence of the transcription factor BATF3, a notable reduction in the presence of CD103+DCs in the lungs and CD8α+ cDC1 in the spleen has been observed.

DCs in lung tissue can be divided into two major subsets: pDC and cDC. The cDC subset can be further divided into XCR1+CD103+cDC1 and SIRPα+CD103− cDC2 (106–110). The functional capabilities of lung CD103+DCs have been a focal point of research, particularly their proficiency in cross-presenting antigens via MHC Class I molecules, which is pivotal for the activation of cytotoxic T lymphocytes (CTLs) during viral infections. *In vitro* and *in vivo* studies have elucidated the role of lung CD103+ DCs in CTL activation (109, 111), highlighting their ability to capture viruses and phagocytose virus-infected apoptotic cells for efficient antigen presentation (105, 112, 113).

CD103+ DCs can phagocytose apoptotic cells not only in the spleen (100) and skin (114), but also in lung tissue (115), further confirming to their specialized phagocytic apoptotic cells function across different tissues. Research led by A. Nicole Desch investigated the selective phagocytic apoptotic cells behavior of lung tissue CD103+cDC1 subsets, Subsequent research further unveiled their reliance on the Mertk receptor for the engulfment of apoptotic cells (116). This specificity distinguishes CD103+cDC1 subsets from other DC subsets, such as CD103-cDC2, in their ability to recognize and phagocytose apoptotic cells.

Extending the exploration to the intestinal, studies have confirmed that CD103+ cDC1 subsets in the inherent layer of the intestines also have the ability to engulf apoptotic cells (117, 118). further validating the concept that specific DC subsets are endowed with distinct functionalities tailored to their anatomical and immunological context.

This growing body of evidence highlights the intricate specialization within the DC lineage, emphasizing the importance of understanding these nuances to unravel the complexities of the immune system’s orchestration of responses to pathogens, tumors, and self-antigens.

### 3.4 pDCs

pDCs are a unique type of DC, known for their ability to produce large amounts of type I interferons, such as interferon-alpha and interferon-beta, particularly in response to viral infections (119). pDCs play a crucial role in the immune system, not only in rapidly responding to viral infections but also in regulating autoimmune responses and other inflammatory reactions (120).

While pDCs do not directly engage in the phagocytosis of apoptotic cells. they play a crucial role in the induction of regulatory CD4+ T cells mediated by efferocytosis. This process is primarily facilitated through the interaction of pDCs with TGF-β, a cytokine released by macrophages that have phagocytosed apoptotic cells. If pDCs are absent, efferocytosis cannot effectively induce the differentiation of regulatory CD4+ T cells, highlighting the indirect yet vital role of pDCs in immune regulation (121).

## 4 Unveiling the receptors: recognition of apoptotic cells by DCs during efferocytosis

In the intricate process of efferocytosis, phagocytes rely on specific surface receptors to identify and ingest dying cells. Numerous systems capable of recognizing phosphatidylserine exist, but a specific phagocytic cell might not always express or utilize all of these systems (35, 122). Although extensive research has delineated various efferocytosis receptors in macrophages, our comprehension of the receptors and underlying molecular mechanisms within DCs remains significantly limited. The comprehensive list of efferocytosis receptors found in DCs and macrophages, along with their corresponding ligands, is detailed in **Table 1**.

**Table 1:** Efferocytosis receptors in dendritic cells and macrophages and their corresponding ligands.

Efferocytosis receptors		Corresponding “eat-me” ligand	Efferocytosis receptors of DCs	Efferocytosis receptors of macrophages	Ref
**TIM**	Tim1	PtdSer	Tim3, Tim4	Tim1, Tim4	(123–126)
	Tim3				
	Tim4				
**TAM**	Tyro3	Gas6, Protein S	Tyro3, Axl	Tyro3, Axl, Mertk	(127–130)
	Axl				
	Mertk				
**Integrin Family**	αvβ5	Mfge8, Del-1, Ccn1, iC3b	αvβ5, αMβ2 αXβ2	αvβ3, αvβ5, αMβ2 αXβ2	(29, 89, 131–133)
	αvβ3				
	αMβ2 αXβ2				
**Scavenger Receptors**	SR-A1	PtdSer	CD36 SR-F1	SR-A1, SR-B1, CD36, SR-F1	(101, 134–137)
	SR-B1				
	CD36				
	SR-F1				
**CD300 Molecules**	CD300a	PtdSer, phosphatidylethanolamine	CD300f (–)	CD300b, CD300f, CD300a (–)	(138–140)
	CD300b				
	CD300f				
**Stabilin Proteins**	Stabilin-1	PtdSer	NA	Stabilin-1, Stabilin-2	(141–144)
	Stabilin-2				
**LRP1**		Calreticulin	Both		(145–148)
**RAGE**		PtdSer	NA	RAGE	(149–151)
**BAI1**		PtdSer	NA	BAI1	(152)

NA, Not Available.

### 4.1 TAM receptors in DC efferocytosis

The TAM receptor tyrosine kinase family, comprising Tyro3, Axl, and Mertk, plays a pivotal role in the recognition of apoptotic cells. These receptors, through their ligands GAS6 or Protein S, indirectly detect phosphatidylserine on apoptotic cells, facilitating efferocytosis. However, the contribution of TAM family members to efferocytosis varies across phagocytes. In macrophages, Mertk deletion completely impairs apoptotic cell clearance, while Axl or Tyro3 deletion, individually or combined, decreases efferocytosis efficiency by approximately 50%. In contrast, bone marrow-derived dendritic cells (BMDCs) and splenic CD11c+DCs predominantly utilize Axl and Tyro3 for apoptotic cell recognition, retaining clearance capability even in Mertk’s absence (127). Notably, a unique DCs subset in the lungs, identified from birth to two weeks, depends on Mertk to effectively clear apoptotic lung epithelial cells (116). This subpopulation, classified as cDC1, expresses intermediate CD103 levels and exhibits a limited capacity for CD8+ T cell proliferation and IFN-γ production induction.

Contradictory findings regarding Axl, Mertk, and Tyro3 expression across DC subpopulations further complicate our understanding. Some studies report Axl and Mertk expression in BMDCs and splenic CD11c+ DCs, excluding Tyro3 (153), while others have identified Tyro3 in a specific BMDC subpopulation marked by CD11c+PDL2+ (154).

The distinct engagement of TAM receptors in DCs compared to macrophages might stem from their unique responses to TAM-specific ligands, Gas6 and Protein S. While Mertk and Tyro3 can be activated by both ligands, Axl responds solely to Gas6. Macrophages, capable of expressing both Gas6 and Protein S, activate all TAM receptors, unlike DCs, which minimally express Gas6. This disparity could underpin the less efficient efferocytosis observed in DCs, though recombinant Gas6 supplementation in mice has been shown to boost efferocytosis by activating Axl (155).

The selective receptor usage may reflect the divergent functional roles within the immune system: macrophages as proficient phagocytes and DCs as specialized antigen-presenting cells, highlighting the complexity and specificity of immune responses during apoptotic cell clearance.

### 4.2 TIM receptors in DC efferocytosis

Beyond the TAM receptor family, the T cell immunoglobulin and mucin domain (TIM) gene family members, including TIM-1, TIM-3, and TIM-4, are instrumental in DC efferocytosis through their ability to bind PtdSer on apoptotic cells, facilitating their clearance (123–125). TIM-3 and TIM-4, in particular, are significant for their efferocytosis roles in DCs. TIM-3, expressed on Th1 and Tc1 cells, is also found on specific DC subpopulations such as CD8+ DCs. TIM-4, both expressed on DCs and macrophages, plays a crucial role in the engulfment of apoptotic cells (156, 157).

Antibody-mediated TIM-3 blockade results in an accumulation of apoptotic cells within mouse splenic follicles and an increase in serum levels of dsDNA antibodies, suggesting a disruption in normal efferocytosis and potential implications for autoimmunity. The interaction between HMGB1 and TIM-3 is pivotal for DCs’ internalization of extracellular dsDNA (158).

The interference in TIM-4 and PtdSer interaction, particularly following dexamethasone-induced thymocyte apoptosis, compromises DC efferocytosis and may predispose mice to autoimmune disorders (125). This indicates the critical role of TIM-4 in maintaining immune tolerance through efficient apoptotic cell clearance. Notably, TIM-4’s recognition of PtdSer does not trigger downstream signaling pathways (159), emphasizing its primary function in the physical engulfment of apoptotic cells rather than initiating intracellular signaling cascades.

### 4.3 Integrin, scavenger and CD300 receptors in DC efferocytosis

DC efferocytosis involves a diverse array of receptors beyond the TAM and TIM families, including integrins αvβ5, the scavenger receptor family member F1 (SR-F1) CD36, and CD300f from the CD300 family. These receptors play distinct roles in the identification and engulfment of apoptotic cells, reflecting the nuanced regulatory mechanisms of efferocytosis in the immune system.

Integrins αvβ5 and CD36 are predominantly expressed in immature DCs, with their expression levels diminishing as DCs mature (89). The αvβ5 integrin, through its interaction with Thrombospondin-1(TSP-1), uniquely contributes to DC efferocytosis (29). The β2 integrin family members αMβ2 and αXβ2 are also involved in the recognition and uptake of apoptotic cells by DCs. This process is achieved through their binding to the complement fragment iC3b on the surface of apoptotic cells (131). The role of αvβ3 integrin in DC efferocytosis has been contested (29, 132), with initial findings suggesting its involvement being later questioned due to the complexity of receptor interactions and the low expression levels of αvβ3 on DCs. Current views suggest that the αvβ3 integrin may only be involved in the efferocytosis of macrophages (160). This specificity underlines the tailored engagement of various integrins in apoptotic cell clearance across different cell types.

Despite CD36 high expression in CD8+ DCs, its necessity for apoptotic cell engulfment remains debatable. Comparative studies of efferocytosis rates between wild-type and CD36-deficient mice DCs show negligible differences (134), suggesting that CD36 may not be critical for DC efferocytosis. This observation contrasts with the role of CD36 in macrophages (135). However, subsequent research has found that CD36 plays a significant role in the efficiency of efferocytosis in DCs (101). With increased CD36 expression, the ability of DCs to engulf apoptotic cells significantly enhances. This finding not only provides new insights into the function of CD36 in the efferocytosis process of DCs but also corroborates its known role in promoting the clearance of apoptotic cells in macrophages (135), indicating that CD36 has a consistent functional across different types of phagocytes.

SR-F1, shared between macrophages and DCs, facilitates DC efferocytosis by recognizing PtdSer through C1q. The absence of SCARF1 leads to efferocytosis impairment, accumulation of apoptotic cells, autoantibody production, and lupus-like disease development in mice (161, 162), indicating its crucial role in maintaining immune homeostasis.

The CD300 family member CD300f, capable of recognizing phosphatidylserine (PS), exhibits divergent effects in different phagocytes—enhancing efferocytosis in macrophages while inhibiting it in DCs (138, 163). This dichotomy further illustrates the complex regulatory landscape of efferocytosis and its dependence on the specific cellular context.

## 5 Interconnected roles of DC efferocytosis and antigen cross-presentation

DCs, as pivotal antigen-presenting cells (APCs), orchestrate the immune response by presenting antigens to T cells. Direct presentation involves APCs displaying exogenous antigens from pathogens like bacteria, viruses, and fungi through MHC class II molecules to CD4+ T cells, and endogenous antigens through MHC class I to CD8+ T cells. Contrarily, cross-presentation allows APCs, predominantly DCs, to present exogenous antigens on MHC class I molecules (164), a critical function for activating CTLs against virus-infected or neoplastic cells.

Efferocytosis is intricately linked to antigen cross-presentation (165). DCs are adept at cross-presenting antigens from apoptotic cells (166), requiring minimal cell quantities and brief exposure periods (3-12 hours) to form MHC-antigen complexes that activate CTLs (87). Furthermore, apoptotic cells serve not only as a rich antigen source for DCs cross-presentation but also as preferred targets (87, 90, 165, 167, 168), emphasizing the significance of efferocytosis in adaptive immunity.

Two recognized pathways facilitate antigen cross-presentation (169): the vacuolar pathway, where antigen processing and MHC class I loading occur within APC phagosomes-endosomes (170–172), and the proteasome-dependent cytosolic pathway, involving exogenous antigen translocation to the cytoplasm for proteasomal processing (173, 174). DCs, especially when compared to macrophages, exhibit superior efficiency in processing and presenting antigens from apoptotic cells via cross-presentation (87), attributed to the unique properties of cDC1 and DNGR-1 receptor actions. The relatively low concentration of lysosomal proteases in DCs limits their ability to degrade proteins, resulting in a significant slowdown of acidification in the effero-lysosomes within DCs compared to macrophages (175). This prolongs the maturation time of effero-lysosomes, creating conditions for long-term antigen retention and effective presentation (176). DNGR-1 (also known as CLEC9A) is a specialized receptor for antigen cross-presentation. It preferentially locates and accumulates in poorly degradable early phagosomes (including effero-lysosomes), without affecting phagosome maturation. DNGR-1 can recognize F-actin-myosin complexes exposed in the effero-lysosomes cavity, and further activate SYK kinase and NADPH oxidase, leading to effero-lysosomal membrane damage and phagosome rupture (177). This process causes the contents of the phagosome to leak into the cytosol, and antigens can be processed and presented via the MHC class I pathway (178). A study identified that effero-lysosomes in macrophages lack MHC class II molecules and related molecules responsible for transporting them from the endoplasmic reticulum-Golgi complex to lysosomes (72), using liquid chromatography-mass spectrometry technology (this study did not perform mass spectrometry analysis on DCs’ effero-lysosomes). However, another study targeting DCs revealed that Rab39a exists in DCs (179). This molecule can transport MHC-I molecules from the endoplasmic reticulum to effero-lysosomes and promote effero-lysosomes to transform into the peptide loading area of MHC-I molecules.

*In vitro* studies have shown DCs capable of cross-presenting antigens from apoptotic cells infected with various pathogens, thereby activating CD8+ T cells (87). This phenomenon has been corroborated by *in vivo* studies, specifically, after injecting apoptotic vesicles generated from BCG-OVA infection into the footpads of mice, researchers observed significant proliferation of specific CD8+ T cells in the cervical lymph nodes of these mice (180). Further *in vitro* studies expanded upon these findings, showing that DCs also cross-present antigens from cells apoptotic due to infections with a range of pathogens including Salmonella enterica serovar Typhi, human cytomegalovirus, vaccinia virus, Mycobacterium tuberculosis, herpes simplex virus (HSV), and Human Immunodeficiency Virus Type 1 (90, 167, 181–184). These studies illustrate the breadth of the cross-presentation phenomenon and stress its significance in immune defense mechanisms.

Progress has been made in understanding the mechanism of antigen cross-presentation following phagocytosis of apoptotic cells. The phagocytic receptors on DCs, Axl, in conjunction with low-density lipoprotein receptor-related protein 1 (LRP1) and RAN binding protein 9 (RANBP9), collectively participate in the internalization of cells undergoing apoptosis post-HSV-1 infection. highlighting the role of phagocytic receptors like Axl, LRP1, and RANBP9 in facilitating antigen cross-presentation post-apoptotic cell engulfment (145). In addition, Annexin1, a protein on the membrane of apoptotic cells, TIM3 (124) and αvβ5 (89) also plays a pivotal role in regulating the efferocytosis and antigen cross-presentation process of DCs (180). The absence of Annexin1 leads to a reduction in LC3-II levels, thereby affecting the processing of antigens within lysosomes and restricting the loading of antigens onto MHC-I. This limitation weakens the capability of DCs to cross-present antigens (180). Tim-3 suppresses MHC-I expression through inhibiting the STAT1-NLRC5 signaling pathway, an effect that has been validated in macrophages (185). However, in a mouse model of acute graft-versus-host disease, Tim-3 expression is notably upregulated in splenic and hepatic T cells, DCs, and macrophages. Although the original studies did not directly explore the relationship between Tim-3 and antigen cross-presentation, its role in modulating the activity of CD8+ T cells suggests that it might indirectly enhance DC-mediated antigen cross-presentation by affecting the functions of T cells and DCs (186). As for integrin αvβ5, while it has been proven essential in the cross-presentation process of DCs (89), the specific mechanisms involved remain unclear and require further investigation.

In addition, the mechanisms by which certain pathogens evade host immune attacks are related to the efferocytosis of DCs. Research led by Kaufmann and his colleagues has shown that when DCs clear apoptotic bodies produced by macrophages infected with Mycobacterium tuberculosis or the BCG vaccine through efferocytosis, they can activate CD8+ T cells, thereby eliciting an adaptive immune response against pulmonary tuberculosis (184, 187). However, Mycobacterium tuberculosis can evade the efferocytic action of DCs by inhibiting the apoptosis of infected macrophages and promoting their necrosis, thus evading the host’s immune response (188–190). Studies have demonstrated that increasing the number of macrophages infected by Mycobacterium tuberculosis and undergoing apoptosis can enhance the efferocytosis of DCs, thereby triggering an early protective immune response against tuberculosis in the body (191).

In the physiological state, the cross-presentation of self-antigens by DCs leads to abortive proliferation of CD8+ T cells (192–196), whose quantity and function are crucial for maintaining the organism’s peripheral tolerance (197).

In conclusion, DC efferocytosis and antigen cross-presentation are fundamental processes that bridge innate and adaptive immunity. Understanding the intricate mechanisms underlying these processes provides insights into the development of novel immunotherapies and vaccines.

## 6 Impact of efferocytosis on DC maturation and function

The maturation of DCs is pivotal for their ability to perform immune functions effectively. Initially, in their immature form, DCs roam the organism, scouting for PAMPs and damage-associated molecular patterns (DAMPs), using their surface pattern recognition receptors (PRRs), such as TLRs. The recognition of these molecular patterns activates DCs and initiates their maturation process (198). During maturation, DCs enhance the expression of major histocompatibility complex (MHC) molecules and co-stimulatory molecules (like CD80 and CD86) on their surface, and they release substantial quantities of pro-inflammatory cytokines, such as interleukin-12 (IL-12), interleukin-6 (IL-6), and tumor necrosis factor-alpha (TNF-α). These cytokines are vital for T cell activation, preparing the immune system to respond to pathogens.

Nevertheless, the journey of DC maturation, especially following the efferocytosis of apoptotic cells, introduces a layer of complexity in their functional dynamics. This process involves nuanced mechanisms that not only influence T cell activation but also play a crucial role in determining whether an immune response will be activated or whether tolerance will be induced. The interplay between efferocytosis and DC maturation is intricate, reflecting the sophisticated regulation within the immune system to maintain a balance between defense and tolerance.

### 6.1 Inhibitory effects of efferocytosis on DC maturation and function: a self-limiting mechanism

The detailed information on the impact of efferocytosis on DC maturation involved in this review is exhibited in **Table 2**. Research shows that under steady-state conditions, the maturation of iDCs is inhibited after they engulf apoptotic cells, primarily characterized by reduced expression of key co-stimulatory molecules such as CD40, CD86, and MHC-II. Even when subsequently stimulated with high concentrations of LPS, these DCs are still difficult to induce to mature (131, 194, 199, 206) indicating that the efferocytosis process can inhibit not only the maturation of DCs but also inflammatory responses.

**Table 2:** The impact of efferocytosis on dendritic cell maturation.

	Source of apoptotic cells	Method of apoptosis induction	Coculture of apoptotic cells and DCs (ACs:DCs)	Impact on DC function	Disease model	Ref
**Efferocytosis inhibits DC maturation**	Mouse monocytes	400ng/mL Staurosporine	4:1 ratio, 5 hr	CD86, MHC-II↓IL-12↑	NA	(199)
	Jurkat T cell line	0.5μg/ml CH1,2.5hr	37°C, 1-2 hr	CD86, MHC-II↓ CCR7↑ CCR2↓, CCR5↓	NA	(131)
	NIT-1 cell line	UV irradiation (10 mJ/m2),1 hr	1:3 ratio, 2 hr	Impaired autologous T cell proliferation	Autoimmune diabetes	(200)
	Mouse syngeneic TAP-KO splenocytes	Osmotic shock	NA	No changes in MHC II, CD40, CD80, and CD86 expression ineffective T cell proliferation	NA	(194)
	Human neutrophils	Aging overnight in Iscove’s medium	2:1 ratio, 2 hr	CD86, IL-12↓ Reduced T cell proliferative capacity	Sepsis	(201)
**Efferocytosis promotes DC maturation**	12B1-D1 cell line	First heat shocked for 1 hr at 42°C, and then Dimerizer AP20187	1:1 ratio, 6 hr	IL-12↑ Enhanced anti-tumor response	Tumor	(202)
	BL6-10 melanoma cell line	20 μM lovastatin,1-2 days	3:1 ratio, 18 hr	CD11b, CD40, CD86↑ Proinflammatory cytokines↑ Chemokines↑ CCR7↑ CCR2↓, CCR5↓	Tumor	(203)
	E.coli infected RAW 264.7 cell line	UVC radiation (0·35 J),4 hr	1:3 ratio, 13 hr	CD86,CCR7↑ IL-1β, IL-10, PGE2↑	NA	(204)
	Neutrophils and A20 B-cell line	0.5 μg/m anti-CD95 antibody 4 hr UVC radiation (0·35 J), 4 hr	1:2 ratio	IL-23, TGF-β↑ IL-12↓ Promoted TH17 differentiation	Natural Infection and Inflammatory	(205)

ACs, Apoptotic Cells.

NA, Not Available.

“↑” indicates increased expression and “↓” indicates decreased expression.

Moreover, this inhibitory effect is limited to those DCs that directly engulf apoptotic cells and does not involve surrounding DCs (201), further emphasizing that it is the direct contact with apoptotic cells that inhibits the maturation of DCs. The latest research corroborates this, showing that after DCs engulf apoptotic cells or lipid-rich nanoparticles, the activation of the LXR pathway, which promotes cholesterol efflux within the cell, plays a significant role in inducing the maturation of steady-state DCs into tolerogenic DCs (78). In studies of autoimmune diseases, it has been found that PGE2 produced by DCs after engulfing apoptotic pancreatic cells significantly inhibits the maturation of DCs and also significantly reduces their ability to stimulate T cell proliferation, thereby preventing the occurrence of autoimmune diseases (200).

Research indicates that prostaglandin E2 (PGE2) not only inhibits the activity and proliferation of CD4+ and CD8+ T cells by promoting the expression of co-inhibitory molecules PD-1 and TIM-3 on T cells (207–209), but also regulates the differentiation and activation of Th17 cells through prostaglandin receptor EP2- and EP4-mediated signaling, cAMP pathways, and by inhibiting the function of transcription factor IRF4 (208, 210). PGE2, through its dual regulation of DCs and T cells, not only reduces the production of inflammatory mediators but also decreases T cell-mediated inflammatory responses, thereby helping to maintain immune homeostasis and prevent the overactivation of autoimmune responses.

Furthermore, surface molecules on apoptotic cells, such as thrombospondin-1 (TSP-1) (199), cytokines secreted by apoptotic cells, such as high mobility group box 1 (HMGB1) (211), and complement fragments like iC3b produced after the activation of the complement system by apoptotic cells (131, 212, 213), also play significant roles in inhibiting the maturation of DCs and promoting immune tolerance.

The immaturity of DCs implies that they are unable to activate specific T cell immune responses or effectively promote T cell proliferation. Although studies have indeed shown that DCs that have phagocytized apoptotic cells can promote the proliferation of CD8+ T cells, more critically, these CD8+ T cells do not show an upregulation of CD25 (a marker indicative of T cell activation) nor a downregulation of CD62L (a molecule crucial for lymphocyte homing and migration). This indicates that despite the observed proliferation of T cells, these CD8+ T cells are neither activated nor prepared to migrate to sites of inflammation or lymphoid tissues. Additionally, although T cell proliferation is observed within the initial 2-3 days, by the 10th day, without additional stimuli to further their maturation, the numbers of these proliferating T cells gradually dwindle (194).

Meanwhile, although the maturation of DCs is inhibited by efferocytosis, they are still able to migrate to secondary lymph nodes after engulfing apoptotic cells in a steady state. After efferocytosis, there is a significant increase in the expression of CCR7 on the surface of DCs. CCR7, a crucial chemokine receptor responsible for guiding DCs to draining lymph nodes, sees increased expression following DC efferocytosis, promoting the migration of steady-state DCs, closely associating with the induction of self-antigen tolerance (214).

**Figure 3:**
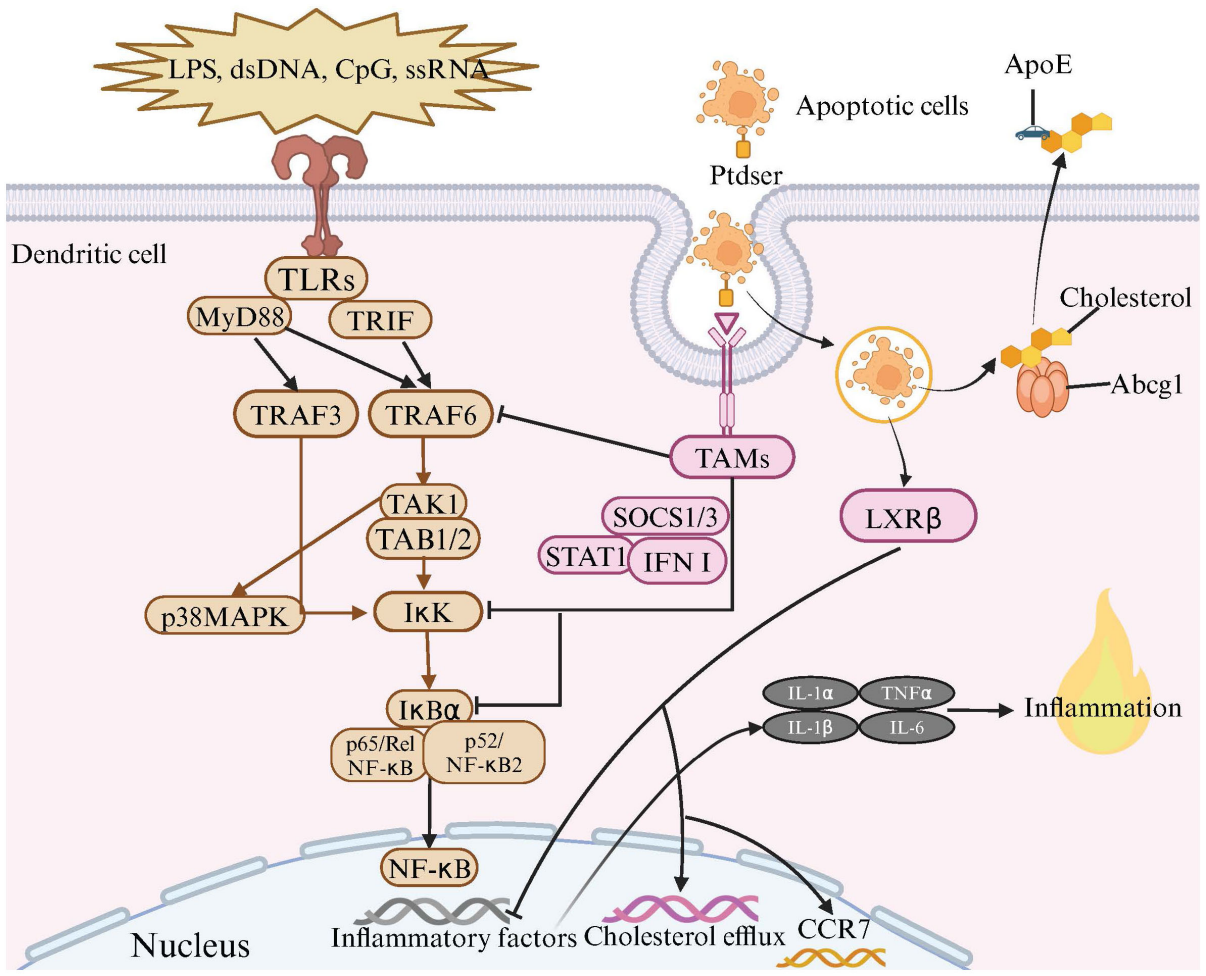
The Anti-Inflammatory Effects of TAM Receptor and LXR Signaling Pathways in Dendritic Cell Efferocytosis. Toll-like receptors (TLRs) are located on the cell membrane and are capable of recognizing various molecules such as lipopolysaccharides (LPS), double-stranded DNA (dsDNA), CpG, and single-stranded RNA (ssRNA). The binding of these molecules to TLRs triggers a series of signal transduction pathways, activating a set of proteins including MyD88, TRIF, TRAF3, TRAF6, TAK1, TAB1/2, and IκK. During this process, the IκK complex phosphorylates IκB protein, leading to its degradation and the release of NF-κB. Subsequently, NF-κB enters the cell nucleus and initiates the transcription of inflammatory factors. Meanwhile, when dendritic cells (DCs) recognize and bind to apoptotic cells through TAM receptors and bridging molecules, TAMs receptors are activated. The activated TAMs receptors, with the assistance of SOCS1/3, STAT1, and type I interferons, inhibit the inflammatory signaling induced by TLRs activation. This includes suppressing the activation of TRAF3/6 and IκK, as well as the degradation of IκBα, thereby inhibiting the NF-κB signaling pathway and preventing excessive inflammation. Upon the engulfment of apoptotic cells by DCs, the LXRβ is activated, leading to the upregulation of genes involved in cholesterol efflux, notably Abcg1 and ApoE. This upregulation facilitates the efflux of cholesterol from the engulfed apoptotic cells to the extracellular matrix. This mechanism not only mitigates the accumulation of cholesterol but also suppresses the transcription of pro-inflammatory cytokines. Additionally, activation of the LXR signaling pathway promotes the transcription of CCR7, enhancing the migration capabilities of tolerogenic dendritic cells, thus contributing to the maintenance of immune homeostasis.

The inability of DCs to mature even when stimulated with LPS following efferocytosis indicates that this process plays a role in regulating TLR-mediated inflammatory responses. Normally, TLR activation by various ligands triggers signaling cascades involving the MAP kinase pathway, NF-κB pathway, and activation of interferon regulatory factors (IRFs), leading to the production of pro-inflammatory cytokines such as IL-6, IL-12, TNF-α, and type I interferons (IFNs) (215). However, DC efferocytosis has been shown to alleviate TLR-mediated inflammatory responses (201, 216) (**Figure 3**), characterized by a reduction in pro-inflammatory cytokine secretion (217). Specifically, DC efferocytosis inhibits the activation of the NF-κB pathway (161, 212), which is central to the inflammatory response and includes members such as p65 (RelA), RelB, cRel, p50, and p52. Under resting conditions, NF-κB is inhibited by IκB proteins, which prevent its nuclear translocation. Upon activation by LPS stimulation, the IKK complex phosphorylates IκB, leading to its degradation and subsequent nuclear translocation of NF-κB to initiate transcription of inflammatory genes. BMDCs pre-treated with apoptotic cells show significant inhibition of IκK activation and IκB degradation upon LPS stimulation, ultimately resulting in restricted nuclear translocation of the nuclear factors cRel, p50, and p65 (212, 218). This inhibitory effect has been shown to depend on the TAM receptors, and in experimental conditions lacking the Mertk gene or employing an antagonistic antibody targeting Mertk, the inhibitory effect of efferocytosis on the NF-κB pathway is absent. The inhibitory role of TAM receptors on inflammation also includes their suppression of the ubiquitination of key TLR signaling components such as TRAF3/6 (153), which is crucial for activating the MAPK and NF-κB pathways in DCs (219).

In mice, the lack of Tyro3, Axl, and Mertk genes leads to an enhanced reactivity of DCs to TLR agonists, resulting in the overproduction of type I interferons and excessive activation of the immune system, causing the mice to die early from systemic inflammatory responses (153). The use of TAM receptor agonists can extensively inhibit cytokine production triggered by TLR 3, 4, and 9, effectively blocking the TLR-induced signaling cascade, particularly the phosphorylation of key signaling molecules ERK1/2 and p38 MAP kinase, as well as the degradation of IκBα and IκBβ (153).

This is attributed to TAM receptors promoting the production of cytokine signaling suppressor proteins SOCS1 and SOCS3 (153). In the presence of STAT1 and type I interferons, these SOCS proteins can effectively limit the IFNγ signaling pathway and reduce the transcriptional activity of NF-κB (220, 221).

By studying how DCs activate specific T lymphocyte responses through efferocytosis, we discovered that DCs do not necessarily need to be fully mature to activate effector T cells (222), challenging the traditional view that directly links maturation state with activation capacity. For instance, comparisons between LPS-treated and untreated DCs on the proliferation of OT1/OT2 T cells *in vivo* demonstrate that while LPS-treated DCs more effectively promote T cell proliferation, untreated DCs also have a significant effect (223). Further research indicates that under normal conditions, Langerhans cells (LCs) can present self-antigens to lymph nodes, which triggers the expansion of antigen-specific CD8+ T cells, rather than fostering tolerance (224). Additionally, Langerhans cells from the skin of K14-mOVA transgenic mice can significantly induce OT-I T cell proliferation even without additional antigen processing (225). Other studies show that CD11c+ CD8α− DCs continuously capture and present gastric self-antigens, such as H+/K+-ATPase, in the absence of an overt inflammatory response, yet fail to completely eliminate or suppress all autoreactive T cells (226), highlighting deficiencies in the establishment and maintenance of self-tolerance. These findings collectively demonstrate the immunological activation capabilities of immature DCs under specific conditions, challenging the previous notion that immature DCs are primarily associated with immune tolerance.

Interestingly, in some cases, mature DCs may also induce T cell tolerance (117, 227), adding complexity to the role of efferocytosis in immune regulation. A comparison of the effects of long-term and short-term exposure to LPS and IFN-γ on human monocyte-derived dendritic cells (moDCs) reveals significant differences in cellular responses. Under long-term activation, DCs not only begin to undergo apoptosis, but also, through involving their surface TAM receptors and efferocytosis, significantly increase their production of IL-10. In contrast, during short-term activation, levels of IL-12p70 and IL-10 are positively correlated, whereas a reverse correlation is evident with long-term activation. Short-term activation sees DCs as the main producers of IL-12p70, but with prolonged activation, DCs become the primary cells undergoing apoptosis and releasing IL-10. Furthermore, DCs that are long-term activated lose their ability to activate allogeneic IFNγ-responsive T cells found in the peripheral blood mononuclear cells of healthy volunteers. Additionally, a multiplex analysis of protein patterns in DC supernatants identifies a specific pattern associated with apoptosis, cancer, and chronic inflammation. This pattern partly overlaps with the well-documented non-functional, tumor-promoting, and anti-inflammatory phenotype of DCs (228).

In summary, DCs that are activated over the long-term exhibit significant functional shifts, evolving towards a non-functional, tumor-promoting, and anti-inflammatory phenotype. These findings underscore the hypothesis that in some cases, mature DCs may also induce T cell tolerance. This nuanced understanding of DC functionality suggests a delicate balance in the immune system, where the context and nature of DC maturation and activation play crucial roles in dictating the outcome of immune responses.

### 6.2 Promotive effects of efferocytosis on DC maturation and function: an enhancing mechanism

As mentioned above, apoptotic cells generally do not possess immunogenicity and their ingestion by DCs usually results in the suppression of DC maturation and triggers an immune tolerance response. However, when DCs ingest apoptotic cells infected with bacteria or viruses, they are often induced to mature (87, 204, 205). This is because when cells undergo apoptosis due to pathogen infection, components of the pathogen (including PAMPs) may adhere to or be included within these apoptotic cells. When DCs ingest apoptotic cells containing PAMPs, they not only take up the cellular debris but also the pathogenic PAMPs (180). DCs recognize these PAMPs through their surface pattern recognition receptors, initiating a cascade of signaling events that lead to DC maturation. However, we have observed that DCs’ tolerance to some apoptotic tumor cells is lost, inducing DC maturation and activating tumor-specific CD8+ T cell immune responses. This finding differs from the usual induction of immune tolerance by apoptotic cells under steady-state conditions. For instance, in the absence of PAMPs, after ingesting apoptotic melanoma cells, BMDCs undergo a series of changes, including increased expression of co-stimulatory molecules (MHC- II, CD40, CD86), pro-inflammatory cytokines (IL-1β, IL-6, TNF-α), and the chemokine receptor CCR7, which promote DC maturation and effectively activate specific T cell responses against melanoma (203). Additionally, studies have compared the effectiveness of co-culturing DCs with apoptotic tumor cells, tumor cell lysates, and supernatants of apoptotic tumor cells in inducing strong anti-tumor immune responses. The results show that co-culturing with apoptotic tumor cells significantly outperforms the other methods in eliciting robust anti-tumor immune responses (229).

This leads us to explore two critical questions: First, why can apoptotic cells from tumors induce DC maturation even without additional stimuli? Second, why can tumor cells in an apoptotic form stimulate DCs to trigger a stronger anti-tumor T cell immune response?

To address the first question, we must consider the unique properties of apoptotic cells from tumors. The enhanced immunogenicity of these cells is believed to be the key reason for the loss of DCs’ tolerance. This is primarily due to the translocation of calreticulin to their surface, increased secretion of HMGB1, and elevated expression of heat shock proteins (HSPs) (230–233).

Calreticulin is typically found in the endoplasmic reticulum of normal cells, where it assists in the proper folding of proteins. However, calreticulin is also abundantly expressed on the surface of tumor cells and further translocates to the cell surface during the apoptosis of tumor cells (231). Research has found that the recognition and uptake of apoptotic tumor cells by DCs depend on the binding of calreticulin on the apoptotic tumor cell surface to its receptor LRP1, which can activate tumor-specific CD8+ T cells (234). Although PtdSer is also present on the surface of apoptotic tumor cells and other efferocytosis receptors exist on DCs, such as the CD36-PtdSer pathway that mediates the recognition and uptake of apoptotic tumor cells, it is normally inhibited by the presence of the autophagy protein ATG5. This inhibition is actually beneficial for the immune attack against tumor cells (101) Blocking the calreticulin-LRP pathway, even when using anti-CD47 antibodies (which aim to block the “don’t eat me” signal on the surface of tumor cells, making them more susceptible to phagocytosis), impairs the phagocytosis of apoptotic tumor cells and significantly weakens or eliminates the tumor-specific CD8+ T cell immune response. This reveals the crucial role of the calreticulin-LRP pathway in promoting phagocyte uptake of apoptotic tumor cells and in the process of antigen cross-presentation (231). LRP1 also affects the intensity of the tumor-specific CD8+ T cell immune response by influencing the expression levels of the antigen cross-presentation-related gene MHC-I. Studies show that knocking out the LRP1 gene or inhibiting its function significantly reduces MHC-I expression levels, leading to a marked reduction in antigen-specific CD8+ T cell immune responses (235).

The increased secretion of HMGB1 following tumor cell apoptosis also promotes the activation of DCs and the presentation of tumor antigens. This is because HMGB1, acting as DAMPs, activates the TLR4-MyD88 signaling pathway in DCs, which not only promotes DC maturation but is also involved in the antigen processing process (236). Studies show that TLR4 can regulate the acidity of lysosomes, thereby affecting the antigen processing process. In DCs lacking TLR4, not only is the acidity within lysosomes increased, but the fusion rate between lysosomes and phagosomes is also accelerated (236–238), leading to lysosomes tending to degrade antigens rather than presenting them (238, 239). Breast cancer patients with dysfunctional TLR4 alleles experience faster recurrence after radiation and chemotherapy compared to those with normal TLR4 alleles (236, 239, 240), indicating a close association between TLR4 and anti-tumor immune responses.

The increased expression of HSPs on the surface of apoptotic tumor cells can significantly induce the maturation of DCs, thereby activating anti-tumor T cell immune responses (202). Inducing apoptosis in tumor cells and promoting the expression of HSPs on their surface is a target of many anti-cancer drugs or therapies. This is because HSPs, also acting as DAMPs, can bind to TLR2 and TLR4 and activate the MyD88/IRAK/NF-κB signaling pathway via a CD14-dependent mechanism, promoting the transcription of inflammatory factors and effectively activating DCs (241).

Whether apoptotic cells can stimulate the maturation of DCs and subsequently induce specific T cell responses not only depends on the apoptotic cells themselves but also on the local cellular and cytokine environment. For example, in experiments where DCs are co-cultured with apoptotic tumor cells, the expression of CCR7 is induced to increase, which aids in the migration of DCs and thereby promotes the formation of anti-tumor immune responses (242, 243), However, in some cases, despite DCs phagocytosing apoptotic tumor cells, their antigen presentation to CD8+ T cells is suboptimal. Yet, with the help of a mixture of cytokines such as IL-6, IL-1β, TNF-α, and PGE2, DCs can effectively activate CD8+ T cell immune responses (244). This suggests that the cytokine environment plays a key role in regulating DCs’ functions and promoting specific immune responses. Additionally, studies have found that macrophages co-localized with apoptotic tumor cells are predominantly of the anti-inflammatory type (245), which promote the production of anti-inflammatory cytokines, thereby driving DCs towards a tolerant phenotype and ultimately negatively impacting DC-mediated anti-tumor immunity (246).

When analyzing and comparing results from different studies, we must consider the variability in immunogenicity among different tumor cell lines, which mirrors the significant differences observed in human tumors in terms of differentiation, drug sensitivity, and prognosis. The variability in immunogenicity of tumor cell lines may affect the interpretation and conclusions of experiments, thereby impacting the accuracy and general applicability of the research (247). This also explains, to some extent, why in some studies, co-culturing apoptotic tumor cells with DCs did not induce DC maturation or effectively trigger tumor-specific T cell responses (244). Enhancing the immunogenicity of apoptotic tumor cells and restoring their apoptotic activity has become a current anti-cancer strategy (248, 249). The technique of loading specific tumor antigens into apoptotic cells to selectively activate T cell immune responses against those antigens has become an important method in studying tumor immune responses (250).

Turning to the second question, compared to tumor cell lysates, apoptotic tumor cells stimulate a more effective anti-tumor immune response in DCs (229). the ability of apoptotic tumor cells to stimulate a more robust anti-tumor T cell immune response is primarily due to their direct contact with DCs. This contact not only triggers the phagocytosis of these cells by DCs — aiding in their clearance — but also initiates significant immune responses. Specifically, direct contact between DCs and apoptotic tumor cells significantly increases the secretion of IL-12 and the expression of CCR7 (242) (251),. IL-12 is crucial for activating allogeneic T cells and natural killer (NK) cells (229). Moreover, CCR7 is essential for guiding DCs to lymph nodes, which are critical sites for initiating potent immune responses (242). While CD8+T cells are typically favored for targeting tumors, evidence suggests that after DCs phagocytose apoptotic tumor cells, the antigens can still be presented on MHC class II molecules, thereby activating tumor-specific CD4+ T cell responses (101). CD4+ T cells are also essential for anti-tumor responses, as they lead to DC “licensing” and initiation, thereby enhancing cross-presentation to CD8+ T cells (252, 253). Thus, these factors collectively amplify the effect of the anti-tumor immune response. Additionally, some researchers believe that the direct contact between DCs and apoptotic cells might mimic a more natural antigen presentation environment, with DC surfaces loaded with an appropriate density of tumor antigen peptides, which helps to activate CTLs with higher affinity and more specificity (244).

The use of apoptotic cells, loaded with specific tumor antigens on their surface to activate T-cell immune responses against targeted tumors, has emerged as a cutting-edge technology in cancer vaccine research (250). Currently, vaccines prepared from tumor cell lysates often fail to induce effective immune responses, partly because the physical form of antigens affects the efficiency of antigen presentation. Compared to soluble antigens, particulate antigens are more effectively cross-presented to MHC-I molecules (254, 255). Although tumor cell lysates contain various soluble antigens, these antigens are typically internalized through complement receptor-mediated phagocytosis rather than by efferocytosis. This mode of internalization may activate tumor-specific CD4+ or CD8+ T cells. However, the lack of cellular structural integrity and mechanical properties in these lysates prevents the formation of stable multimeric complexes with mechanoreceptors like integrins, which may limit the transmission of mechanical signals during the phagocytosis of tumor cell lysates (256), thereby potentially restricting the immune response to the tumor to a certain extent.

## 7 Efferocytosis on DCs: guardian of immune homeostasis

The immune responses activated by macrophages and DCs following efferocytosis exhibit marked differences. Macrophages exhibit a higher efficiency of efferocytosis compared to DCs and are capable of continuously engulfing and clearing apoptotic cells, often being a primary focus of efferocytosis research. Post-efferocytosis, macrophages secrete anti-inflammatory mediators such as IL-10 and TGF-β, as well as specialized pro-resolving molecules (257), and suppress pro-inflammatory cytokines including TNF-α, IL-1β, and IL-12. They also upregulate genes that enhance the internalization and degradation of apoptotic cells (117, 258, 259), thereby maintaining immune homeostasis. In contrast, DCs, upon undergoing efferocytosis, primarily activate anti-inflammatory pathways through the upregulation of genes involved in the differentiation of regulatory T cells (iTregs) and antigen presentation (117), thereby highlighting the unique anti-inflammatory mechanisms of DCs at the transcriptomic level following efferocytosis. All differences between DCs and macrophages are detailed in **Table 3**.

**Table 3:** Comparison of characteristics and functions between conventional dendritic cells and macrophages.

Feature	cDCs	Macrophages
**Origin**	Derived from bone marrow precursors	Bone marrow CCR2+ monocyte precursors
**Characteristics**	Under steady-state conditions, cDC lifespan ranges from several days to a week; more rapidly depleted during inflammation or infection; Replenished by bone marrow precursors.	Capable of self-renewal, persists long-term in specific tissues or microenvironments.
**Pathways for Pathogen/Antigen Capture**	FcγR-mediated phagocytosis; Non-selective pinocytosis; Efferocytosis mediated by specific receptors such as αvβ5, Tim-3.	Phagocytosis, pinocytosis pathways similar to cDCs; Efferocytosis utilizes multiple receptors to recognize and bind apoptotic cells.
**Antigen Presentation Pathway**	MHC-II pathway, MHC-I pathway (cross-presentation).	MHC-II pathway
**Characteristics of immune surveillance**	Downregulates phagocytic activity after capturing pathogens, apoptotic cells; Strong migration capability to lymph nodes; Activates naive CD4+ T cells and CD8+ T cells.	Continuous phagocytosis of pathogens and apoptotic cells, typically non-inflammatory; No intervention from other immune cells required, Recruits neutrophils and monocyte precursors to inflammation sites during severe infections.
**Antigen Processing Capability**	Mild internal environment, low acidity, weak protease activity; antigen not effectively degraded, antigen information preserved (specific to cDC1).	High lysosomal acidity, strong protease activity, antigen thoroughly degraded.
**Immune Homeostasis**	Promotes Tregs differentiation to maintain peripheral tolerance;	Non-inflammatory phagocytosis and degradation of bacteria; Promptly phagocytoses apoptotic cells to avoid secondary necrosis; Secretes anti-inflammatory cytokines, SPMs to promote tissue repair and resolve inflammation.
**Pathological Significance**	Activates CD8+ T cells in pathogen and tumor microenvironments, aiding in the complete eradication of pathogens and promoting anti-tumor immune responses;	Shifts to M2 type (anti-inflammatory and pro-healing) after phagocytosing apoptotic tumor cells, suppressing immune response and potentially promoting tumor growth; Infection may spread systemically when phagocytosis occurs but pathogens are not effectively degraded.

The phenomenon of DC mediated efferocytosis plays a pivotal role in fostering immune tolerance through the differentiation of iTregs. This process involves DCs engulfing apoptotic cells, subsequently processing and presenting the derived antigens, which is instrumental in establishing and maintaining peripheral T cell tolerance. Unlike central tolerance, which is achieved through the negative selection of autoreactive lymphocytes within the thymus and bone marrow (260, 261), peripheral tolerance is heavily reliant on CD4+ Tregs expressing the transcription factor Foxp3 (262, 263). These include thymus-derived naturally occurring Tregs (nTregs) and peripherally induced iTregs (264, 265).

### 7.1 Fostering immune tolerance and anti-inflammatory responses:enhancing Treg cell proliferation and function

**Figure 4:**
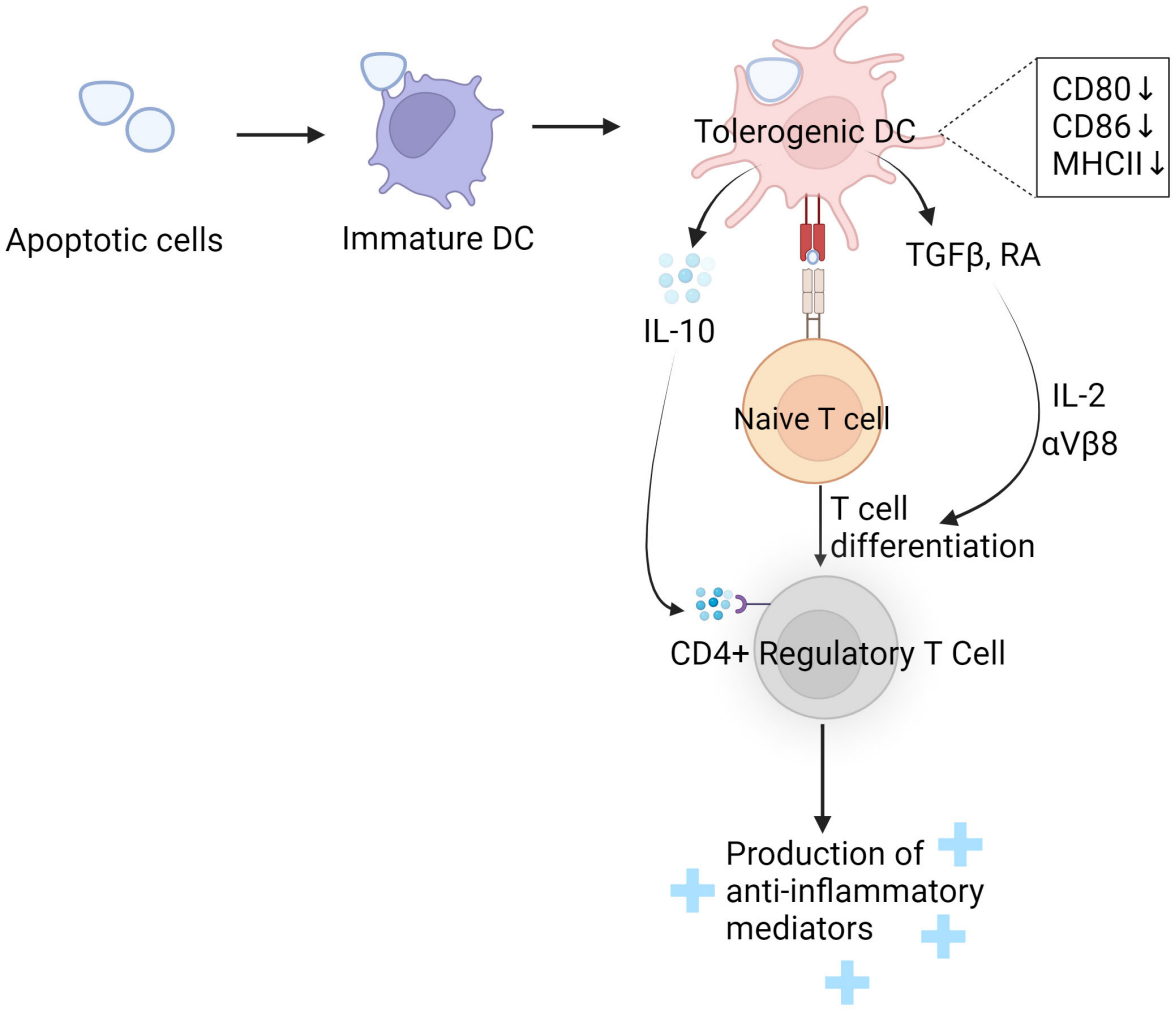
DC phagocytosis induces Treg differentiation and enhances its function. When immature dendritic cells (DCs) engulf apoptotic cells, they undergo a transformation into tolerogenic DCs. This transformation is marked by a reduction in the expression of co-stimulatory molecules. These tolerogenic DCs then produce and release immune-modulating substances such as IL10, TGF-beta, and retinoic acid (RA). The IL10 secreted by tolerogenic DCs binds to IL10 receptors on regulatory T cells (Tregs), which enhances the Tregs’ ability to function effectively. On the other hand, TGF-beta and RA work together in the presence of IL2 and integrins αvβ8 to drive the differentiation of naive T cells into regulatory T cells. These regulatory T cells play a crucial role in establishing and maintaining immune tolerance. They achieve this by releasing anti-inflammatory mediators, which help in preventing overactive immune responses that could lead to autoimmunity or chronic inflammation.

Tregs are essential in dampening pro-inflammatory responses and aiding the resolution of inflammation. The expansion of Treg cells can boost the process of efferocytosis in a significant disease model characterized by both a reduction in Treg cells and impaired efferocytosis (266). Emerging evidence indicates that DC efferocytosis can promote the differentiation of Tregs (228, 267, 268) (**Figure 4**), This establishes an effective positive feedback loop. The mechanism by which DC efferocytosis facilitates Treg differentiation involves both TGF-beta and RA (269–274). DCs serve as significant producers of TGF-β (275). The absence of TGF-β results in the progressive depletion of iTregs within peripheral lymphoid structures (276). Conversely, exogenous TGF-β can trigger Foxp3 expression in specific subsets of T cells (277, 278), pivotal for iTregs formation (279). The importance of this pathway is underscored by observations that mice deficient in Foxp3 or TGF-βRII succumb to early death due to uncontrolled systemic inflammation (280, 281).

The generation of iTregs by TGF-β is highly dependent on the integrin αvβ8 (282–284). In the intestinal context, CD103+ DCs lacking this integrin fail to foster iTregs *in vitro* (285), leading to a marked decrease in iTregs numbers within the colonic mucosa (286). Moreover, RA, a metabolite of vitamin A produced by DCs, along with IL-2 from T cells, further augments TGF-β induced Foxp3 synthesis, enhancing iTreg differentiation (287, 288). This process also contributes to nTregs differentiation within the thymus, although TGF-β’s role in nTregs production was initially debated (289, 290). However, further studies revealed TGF-β’s protective role in safeguarding nTregs from apoptosis during thymic development (291).

In the intestinal mucosal layer, CD103+DCs, post-clearance of apoptotic intestinal epithelial cells, regulate a host of genes pivotal for iTregs proliferation, differentiation, recruitment, and migration, including Ccl22, Ccl17, Aldh1a2, IDO-1, GARP, Cd274 (117).

Additionally, DCs, upon phagocytosing apoptotic cells post-infection, produce substantial amounts of IL-10 (161, 204, 292), which acts through enhancing the suppressive function of Treg rather than promoting their proliferation and differentiation, as TGFβ does (293).

## 8 Conclusion and future perspectives

Despite the significant attention and progress that efferocytosis has garnered in recent research, our understanding remains limited regarding how DCs can precisely guide beneficial immune responses following the recognition and engulfment of apoptotic cells from diverse origins. For instance, in the field of oncology, the role of macrophage-mediated efferocytosis has been extensively documented to be closely associated with tumor immune evasion (128, 294, 295). In contrast, DCs are capable of initiating specific immune responses against tumor cells by engulfing apoptotic tumor cells (101). Compared to DCs, macrophages exhibit proficient efferocytosis and metabolic processing capabilities of cellular debris. However, during Mycobacterium tuberculosis infection, they may contribute to the pathogen’s resistance to host immune responses. In this context, the efferocytosis of apoptotic bodies released by infected macrophages by DCs triggers the host’s adaptive immunity against tuberculosis (184, 187).

This phenomenon underscores the irreplaceable and critical role of DCs efferocytosis in immune responses. But the efferocytosis by DCs is exceedingly complex within immune regulation, involving a multitude of signaling pathways, cooperative actions among different cell types, and diverse interactions between DCs and apoptotic cells. Although we have gained some understanding of how DCs clear apoptotic cells through efferocytosis to maintain tissue homeostasis and prevent uncontrolled inflammatory responses, the mechanisms by which DCs precisely regulate immune responses to promote immune tolerance or activate specific immune responses remain somewhat unclear. This review focuses on the pivotal role of DCs in efferocytosis and how they influence the balance of the immune system through this process. Several problems remain to be resolved in the future:

1. Some efferocytosis receptors, after accomplishing the task of recognizing and engulfing apoptotic cells, continue to participate in the subsequent antigen cross-presentation pathways of DCs, which may be one of the crucial mechanisms linking innate and adaptive immunity. Efferocytosis receptors are not merely facilitating the clearance of dying cells; they may also activate or modulate specific signaling pathways during efferocytosis, thereby influencing the maturation and functionality of DCs, and contributing to the formation of a comprehensive and effective immune response. Future research needs to delve deeper into the specific mechanisms of these efferocytosis receptors and their roles in regulating immune responses. Additionally, there is a need to discover unexplored signaling receptors to comprehensively explain the critical functions of DCs in immune surveillance and defense.
2. Integration of Efferocytosis with DCs’ Tolerance Induction: While the involvement of efferocytosis in antigen cross-presentation by DCs is acknowledged, its role in the induction of immune tolerance remains less understood. How do DCs, following efferocytosis, differentiate between contexts requiring immune activation versus tolerance induction? Identifying the cues and mechanisms that guide this decision-making process is crucial for understanding how efferocytosis contributes to maintaining immune homeostasis and preventing autoimmune diseases.
3. Impact of the Microenvironment on DC Efferocytosis: The microenvironment in which efferocytosis occurs can greatly influence the outcome of this process. Factors such as tissue-specific signals, the presence of inflammatory cytokines, and interactions with other immune cells can modulate DCs’ responses to engulfed apoptotic cells. Future research should explore how these microenvironmental factors affect efferocytosis by DCs and their subsequent immune functions. Additionally, understanding how DCs integrate these signals to orchestrate appropriate immune responses will shed light on their versatility and adaptability in diverse physiological and pathological contexts.

With the advancement of technologies such as spatial transcriptomics and advanced imaging techniques, we now have the opportunity to explore the spatial and molecular dimensions of DC efferocytosis with unprecedented detail and precision. These technologies can reveal how DCs recognize and engulf apoptotic cells within the tissue microenvironment and how this process impacts their antigen presentation and immune regulatory functions. Through spatially resolved transcriptomic analysis, we can gain a deeper understanding of the heterogeneity of DC efferocytosis across different tissues and pathological states. This will aid in uncovering novel regulatory mechanisms of efferocytosis receptors and how they interact with other immune cells and factors within the tissue microenvironment. Consequently, we are encouraged to develop new diagnostic tools and therapeutic strategies aimed at specifically modulating the efferocytosis function of DCs to treat immune-related diseases caused by aberrant efferocytosis.

## Author contributions

YM: Writing – original draft. TJ: Writing – review & editing. XZ: Writing – review & editing. YX: Writing – review & editing. KW: Writing – review & editing. TZ: Writing – review & editing. MX: Writing – review & editing.
